# Differences in Brain Structure and Function Among Yoga Practitioners and Controls

**DOI:** 10.3389/fnint.2018.00026

**Published:** 2018-06-22

**Authors:** Neha P. Gothe, Jessica M. Hayes, Cindy Temali, Jessica S. Damoiseaux

**Affiliations:** ^1^Department of Kinesiology and Community Health, University of Illinois at Urbana Champaign, Champaign, IL, United States; ^2^Department of Psychology and Institute of Gerontology, Wayne State University, Detroit, MI, United States

**Keywords:** cognition, fMRI, mind-body exercise, frontal pole, executive function

## Abstract

**Background:** Yoga is a mind-body based physical activity that has demonstrated a variety of physiological, psychological and cognitive health benefits. Although yoga practice has shown to improve cognitive performance, few studies have examined the underlying neurological correlates.

**Objective:** The current study aimed to determine the differences in gray matter volume of the hippocampus, thalamus and caudate nucleus and brain activation during the Sternberg working memory task.

**Method:** Participants were 13 experienced yoga practitioners (mean age = 35.8), defined as having more than 3 years of regular yoga practice, and 13 age- and sex-matched controls (mean age = 35.7). All participants completed a 6-min walk test to assess fitness, psychosocial and demographic questionnaires; and underwent magnetic resonance imaging to assess gray matter volume and brain activation.

**Results:** There were no group differences on demographic measures of income, education and on estimated VO2max or physical activity levels. Gray matter volume differences were observed in the left hippocampus, showing greater volume in experienced yoga practitioners compared to controls (*p* = 0.017). The functional MRI results revealed less activation in the dorsolateral prefrontal cortex in yoga practitioners compared to controls during the encoding phase of the Sternberg task (*p* < 0.05). Reaction time and accuracy on the task did not differ between the groups.

**Conclusions:** Our results suggest an association between regular long-term yoga practice and differential structure and function of specific brain regions involved in executive function, specifically working memory, which has previously shown to improve with yoga practice. Future studies need to examine intervention effects of yoga and explore its potential to maintain and improve cognitive health across the lifespan through longitudinal and intervention studies.

## Introduction

Yoga, a mind-body activity that has components centering on meditation, breathing and postures has become increasingly popular in recent years. It’s health benefits are being systematically investigated and it is acknowledged today as an effective therapy for a variety of physical conditions such as pain and associated disability (Büssing et al., 2012; Cramer et al., 2013a), arthritis (Haaz and Bartlett, 2011), rheumatic diseases (Cramer et al., 2013b), cardiopulmonary and musculoskeletal function (Raub, 2002) as well as some psychological conditions including depression (Uebelacker et al., 2010) and anxiety (Kirkwood et al., 2005).

In addition to the mounting evidence for the health benefits of yoga, its effects on cognition have become a particularly important area of inquiry in recent years. A systematic review and meta-analysis examined the acute (short term, single bout) and intervention effects of yoga on cognitive function across 15 randomized trials and seven acute studies (Gothe and McAuley, 2015). The effect sizes observed for the different cognitive functions including attention, processing speed, executive functions and memory ranged from g = 0.18 to g = 0.29 for the randomized trials and were even greater in magnitude for acute studies of yoga, ranging from g = 0.39 to g = 0.78. The studies reviewed in this meta-analysis used behavioral measures to assess cognitive function, such as computer- and paper pencil-based tests of executive function, attention, processing speed and memory.

Fewer studies have examined the neurobiological correlates of yoga practice using advanced imaging techniques. Froeliger B. E. et al. (2012) and Froeliger B. et al. (2012) examined structural and functional brain differences in Hatha yoga meditation practitioners and meditation naïve controls. Seven Hatha yoga meditation practitioners were compared with seven matched controls and were found to have significantly larger gray matter volume in the prefrontal cortical regions, including the middle and orbital frontal gyri for the yoga group than the controls. In subcortical regions, yogis were found to have a significantly larger left parahippocampal gyri and hippocampus than controls. A similar influence of yoga was observed in an intervention study, which revealed a bilateral increase in hippocampal size following a 6 month yoga intervention in older adults (Hariprasad et al., 2013). In a second study (Froeliger B. E. et al., 2012; Froeliger B. et al., 2012) the effects of yoga meditation on emotion-cognition interactions were investigated as the same subjects performed an affective Stroop task in the MRI and found that while viewing negative emotional images yoga meditation practitioners displayed less activation in the dorsolateral prefrontal cortex than controls.

Executive function refers to a subset of goal-directed processes such as planning, decision making, working memory, cognitive flexibility, abstract thinking, and has been repeatedly shown to improve with regular erobic exercise (Smith et al., 2010) as well as yoga (Gothe et al., 2014; Gothe and McAuley, 2015). Working memory includes a subset of processes involved in the active encoding, maintenance and manipulation of information, and its retrieval typically following a short period of time (Miyake et al., 2000; Kane et al., 2007). Encoding occurs when information is first perceived while maintenance refers to the retention of that information over a short delay. Retrieval is the process of later recalling that information. In a study by Gothe et al. (2014), an 8 week yoga intervention had the most significant impact on working memory performance among a sample of middle aged and older adults compared to a stretching and strengthening control group. Acute effects of yoga on executive function have also shown to improve performance on working memory and inhibitory control measures (Gothe et al., 2013) following a brief 30 min Hatha yoga sequence. It is unknown, however, if these differences in working memory stem from benefits to a single subcomponent process in working memory (i.e., encoding, maintenance, or retrieval) or from benefits to multiple subcomponent processes.

The purpose of this study was to examine structural and functional differences between experienced yoga practitioners and age- and sex-matched controls. Based on recent evidence from imaging studies showing greater hippocampal volume after yoga practice, we hypothesized finding greater hippocampal volume in yoga practitioners compared to controls. Research from human cognitive studies demonstrates some specificity, such that exercise influences some brain regions like the hippocampus selectively, and has minimal or no influence on others (Hillman et al., 2008; Erickson et al., 2011). We therefore included two brain regions: thalamus and caudate nucleus as controls, to test whether the regional specificity is also observed among long term yoga practitioners. Given that working memory seems to be the domain of cognition that is most significantly impacted by yoga training, the present study also aimed to identify the neural correlates of working memory performance among experienced yoga practitioners using functional imaging techniques. We used the Sternberg task (Sternberg, 1966), which requires participants to encode a series of stimuli into their working memory to decide whether a probe stimulus that is presented at a later time point was present in the encoded series. The task captures the three processes associated with working memory: encoding of information, storage in short term memory, and the retrieval of information in response to the probe. Distinguishing between these three sub-component processes allowed us to pinpoint which specific component(s) showed differences in brain activation between experienced yoga practitioners and controls. Given the known cognitive benefits of yoga, but limited knowledge of its neural correlates, we expected to find different brain activation patterns during the Sternberg task in experienced yoga practitioners compared to controls without a predilection for specific sub-component processes. We also predicted that the experienced yoga practitioners would perform the Sternberg working memory task with greater accuracy than the controls.

## Materials and Methods

### Participants

Participants consisted of experienced yoga practitioners (*n* = 13) and age- and sex-matched controls (*n* = 13). Flyers and advertising brochures were posted around the Detroit-Metro area and participants were recruited by targeting local yoga studios, community centers, and the student and staff list-serv at Wayne State University. Interested individuals were screened and excluded if they had a history of stroke, brain damage, or any significant medical or neurological illness. Left handedness, poor hearing or vision, current use of psychotropic medication, and the presence of MRI contra-indications also served as exclusion criteria. Experienced yoga practitioners were defined as individuals who reported three or more years of yoga experience and regular on-going yoga practice (>3 days per week, at least 1 h per day). Control participants reported no current or past experience with yoga or any other type of mind-body practice. All procedures performed in this study involving human participants were in accordance with the ethical standards of the Wayne State University, Institutional Review Board and with the 1964 Helsinki declaration and its later amendments or comparable ethical standards. Informed consent was obtained in person from all participants in this study.

### Procedures and Measures

Participants completed two lab based visits to participate in the study. Written informed consent and demographic information including age, income, education and marital status was collected during the first visit. All participants completed the self-report Godin leisure time exercise questionnaire (Godin and Shephard, 1985) that assessed typical weekly engagement in physical activity. Experienced yoga practitioners were asked to complete a separate form to gather their yoga history which included—years of yoga training, type of yoga practice (Hatha, Iyengar, etc.), dose of weekly yoga practice and time spent in performing postures, breathing and meditative exercises. Anthropomorphic measures of height and weight were assessed using an electronic stadiometer to calculate body mass index. Because cardiorespiratory fitness has been shown to influence cognitive performance, all participants completed the 6-min walk test (Balke, 1963) validated among healthy adults (Enright and Sherrill, 1998) to estimate peak cardiorespiratory fitness. Independent samples *t*-tests were conducted to check whether the two groups were similar on all demographic and physical characteristics.

### MRI Acquisition

Scan sessions were conducted on a 3 Tesla Siemens Magnetom Verio scanner using a 32-channel Head Matrix coil at the Wayne State University MR Research Facility in Detroit.

A T1 weighted MP-RAGE sequence was used to acquire whole-brain structural images: repetition time (TR) = 1680 ms, echo time (TE) = 3.51 ms, 176 slices, voxel size = 0.7 mm × 0.7 mm × 1.3 mm, flip angle (FA) = 9°, field of view (FOV) = 256 mm. A 10 min and 15 s T2*-weighted gradient-echo sequence was used to acquire functional images: TR = 2200 ms, TE = 30 ms, 37 slices parallel to the AC-PC plane, voxel size = 2.8 mm × 2.8 mm × 2.8 mm, volumes = 276, FA = 80°, FOV = 220 mm.

### Sternberg Working Memory Task

During the functional MRI scan, participants completed a Sternberg working memory task paradigm so that the encoding, maintenance and retrieval subcomponent processes of working memory could be investigated (Sternberg, 1969). Participants practiced the task before the MRI scan session and task instructions were repeated during the scan session. For each of the 40 trials, participants were instructed to remember a set of four upper-case letters that were presented for 2 s. After this initial presentation, a fixation cross was displayed for 2 s, followed by the presentation of a single lower-case probe letter for another 2 s. As has been done in previous studies, the probe letter was the opposite case of the original letter set in order to prevent encoding based only on visual information (Bedwell et al., 2005). Participants were asked to indicate as quickly as possible whether the probe letter was present in the original set of upper-case letters. The 40 trials were evenly split between match and non-match conditions in which the probe letter did or did not match one of the letters in the original stimulus set. The inter-trial interval with a jittered length of 3–5 s followed before the next trial began. The task was presented on an in-bore screen using an Avotec Silent Vision (SV-6011) projection system, which was made visible to the participants through a mirror that was mounted on the head coil. E-Prime 2.0 software was used to program and present the task (Psychology Software Tools, Pittsburgh, PA, USA). The participants provided yes/no responses through a two-button response box that they held in their right hand.

Responses were not recorded for three participants due to technical difficulties, but imaging data from these individuals was retained as the remaining participants showed high accuracy in completing the task (*M* = 0.96, *SD* = 0.04). Task accuracy was calculated as the number of correct yes/no responses out of 40 trials and reaction time was calculated as the average length of time in ms. between probe presentation and responding across the 40 trials for each participant. Independent samples *t*-tests were used to compare accuracy and reaction time between groups using SPSS 23.

### Data Analysis

Image processing and analysis was carried out using FSL 5.0.8 (FMRIB’s Software Library) software tools (Smith et al., 2004). In addition to using FSL’s Brain Extraction Tool (BET; Smith, 2002) to remove non-brain voxels from the structural and functional images, the first five volumes of the functional images were removed. Further preprocessing of the functional images included motion correction with MCFLIRT (Jenkinson et al., 2002), temporal filtering with a high pass filter of 100 s grand-mean intensity normalization, and spatial smoothing using a Gaussian kernel of full width half maximum (FWHM) of 6.0 mm. Functional images were linearly registered first to the high resolution structural scans using boundary-based registration, and then to the standard 2 mm Montreal Neurological Institute (MNI) template using 12 degrees of freedom with FMRIB’s Linear Image Registration Tool (FLIRT; Jenkinson and Smith, 2001; Jenkinson et al., 2002).

### Subcortical Volume and Shape

**Figure 1:**
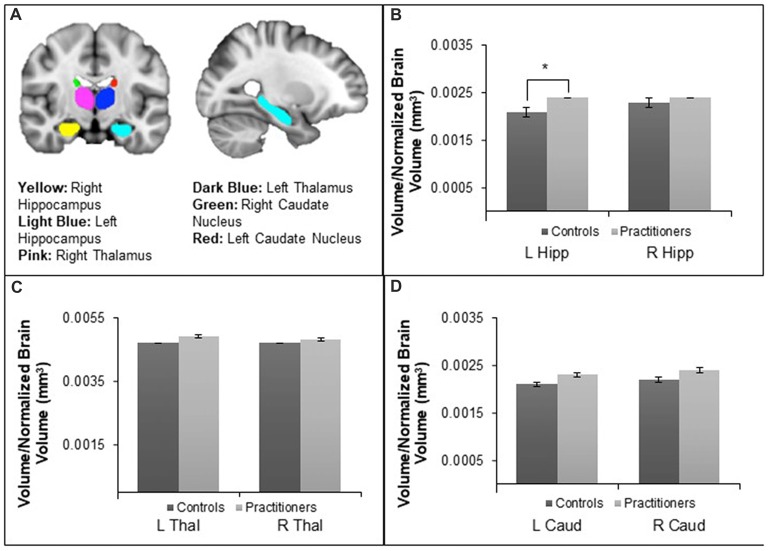
Panel **(A)** depicts the segmentation of the hippocampus, thalamus and caudate nucleus using FSL FIRST. Panel **(B)** shows differences in the left hippocampal (Hipp) volume relative to normalized brain volume between the yoga experts and controls, *t*_(24)_ = −2.571, *p* = 0.017. There were no differences in right hippocampal volume. Panel **(C)** shows that there were no volume differences in the right or left thalamus (Thal). Panel **(D)** shows that there were no volume differences in the right or left caudate nucleus (Caud). *p < 0.05.

FSL FIRST (Patenaude et al., 2011) was utilized to segment subcortical structures for subsequent volumetric analysis, see Figure 1A. Prior to the segmentation of subcortical structures, FIRST performs registration by transforming the T1 images to standard space using an affine transformation with 12 degrees of freedom. Subcortical structures are located following registration by using a sub-cortical mask to exclude voxels outside those regions and then shape models and voxel intensities are utilized to segment structures of interest. After segmenting the bilateral hippocampus, thalamus and caudate nucleus on the T1 structural scans, the command-line utility fslstats was used to report the volume of each of these structures for each individual participant. In order to control for variation in the volume of subcortical structures that may be due to variation in head size, FSL SIENAX was used to obtain an estimate of total brain tissue volume normalized brain for participant head size. All subcortical structure volumes were calculated relative to this normalized brain volume and then the average volumes for the experienced yoga practitioner and control groups were compared using an independent sample *t*-test in SPSS 23. Significance levels for tests of volume differences in structures of interest (left and right hippocampus) were corrected for multiple comparisons using a Bonferroni correction. Thalamus and caudate nucleus served as control regions as we hypothesized there would be no differences between the groups. Vertex analysis using FIRST was conducted to identify localized shape differences in the segmented subcortical structures. Group differences between experienced yoga practitioners and controls in the shape of these structures were investigated on a per-vertex basis with further statistical analyses being run by randomize (Winkler et al., 2014).

### Brain Activation During Working Memory Subcomponent Processes

Both levels of whole-brain analyses used a General Linear Model, as implemented in FSL FMRI Expert Analysis Tool v6.00 (FEAT). Time-series statistical analysis of the first-level data relied on FMRIB’s Improved Linear Model (FILM) with local autocorrelation correction (Woolrich et al., 2001). Encoding was modeled as the 2 s in which the original four letters were presented, maintenance was modeled as the following 2 s in which a fixation cross appeared, and retrieval was modeled as the 2 s in which the singular letter was displayed and participants made their response. A three-column event file specified the timing for each of these three components. Null events, in which a fixation cross was presented, occurred between each complete task trial and were not modeled. These null events therefore served as the baseline condition. The main effects of encoding, maintenance and retrieval were calculated as the difference between activation during each of these subcomponents compared to baseline.

Higher-level analyses using FMRIB’s Local Analysis of Mixed Effects (FLAME) modeling was carried out to determine differences in activation during encoding between experienced yoga practitioners and controls. The resulting z-stat images were masked with a gray matter mask and significance within the gray matter was determined using a *Z* statistic threshold of 2.3 and a corrected cluster threshold of *p* = 0.05 (Worsley, 2001).

## Results

### Participant Characteristics

A total of 26 healthy adults between the ages of 19 and 58 (*M* = 35.73, *SD =* 14.71) resulted in 13 matched pairs for the study. A majority of pairs were females (12/13). In addition to being age- and sex-matched, there were no significant differences in other demographic characteristics between the experienced yoga practitioners and controls as seen in Table 1. Importantly, there were no between group differences in self-reported levels of physical activity or in the estimated levels of physical fitness.

**Table 1:** Demographic and yoga history characteristics of experienced yoga practitioners and controls.

Measure	Yoga experts (*N* = 13)	Controls (*N* = 13)
Age (years)	35.77 ± 15.43	35.69 ± 14.57
Sex (*n* Males/*n* Females)	1/12	1/12
Body mass index	23.52 ± 4.36	26.41 ± 6.76
Godin physical activity score	150.46 ± 80.08	119.84 ± 180.48
Estimated VO2max (mL/kg/min)	35.59 ± 7.29	33.61 ± 8.03
Marital status *(n)*		
Single	6	5
Partnered/Significant other	3	2
Married	2	3
Separated/Divorced	2	3
Education *(n)*		
1–3 years of College	3	5
College/University Graduate	5	4
Master’s Degree	3	2
PhD or Equivalent	2	2
Race *(n)*		
African American	2	4
Caucasian	11	8
Asian	0	1
**Yoga History**
Years of yoga practice	9.31 ± 6.25	N/A
Days/week of yoga practice	4.15 ± 1.77	N/A
Hours/day of yoga practice	4.38 ± 2.57	N/A
% Time spent in yoga postures	66.69 ± 25.74	N/A
% Time spent in yogic breathing	16.38 ± 16.17	N/A
% Time spent in yogic meditation	16.92 ± 13.23	N/A

*Note: No significant group differences were observed on any of the variables (all p’s > 0.05)*.

On average the yoga practitioners had 9.31 years (range of 5–24 years) of yoga experience. They reported practicing yoga on 4.1 days/week for 4.38 h/day. Participants reported engaging in the practice of yoga postures for 66.69% of their time, yogic breathing for 16.38% of their time and yogic meditation for the remaining 16.92% of their practice time. Hatha yoga was the most commonly practice form of yoga (10/13) while three other experts reported Kundalini (1/13) and Iyengar (2/13) as their primary style of yoga practice.

### Sternberg Working Memory Task

Performance on the Sternberg working memory task was measured by calculating accuracy in yes/no responses and by calculating the average reaction time (seconds) in making a response. There was no significant difference (*t*_(21)_ = 1.26, *p* = 0.222) in performance accuracy, as measured by the number of correct yes/no responses out of the 40 trials between experienced yoga practitioners (*M* = 0.95, *SD* = 0.04) and controls (*M* = 0.97, *SD* = 0.03). Similarly, average reaction time to the probe in milliseconds did not differ (*t*_(21)_ = −0.324, *p* = 0.749) between experienced yoga practitioners (*M* = 0.932, *SD* = 0.16) and controls (*M* = 0.907, *SD* = 0.19).

### Neuroimaging

#### Subcortical Volume and Shape

The average volume (mm^3^) of the bilateral hippocampus, thalamus and caudate nucleus relative to the normalized brain volume of each participant was averaged within the yoga practitioners and the control groups and then compared between groups. The average left hippocampal volume of experienced yoga practitioners (*M* = 0.0024, *SD* = 0.00027) was found to be greater than that of their control counterparts after multiple comparison correction (*M* = 0.0021, *SD* = 0.00023) (*t*_(24)_ = −2.571, *p* = 0.017, *d* = −0.85), see Figure 1B. The average volumes of the other subcortical structures, including the right hippocampus (*t*_(24)_ = −0.676, *p* = 0.344), the left and right thalamus (*t*_(24)_ = −0.868, *p* = 0.394 and *t*_(24)_ = −0.453, *p* = 0.654 respectively), and the left and right caudate nucleus (*t*_(24)_ = −1.803, *p* = 0.084 and *t*_(24)_ = −1.789, *p* = 0.086 respectively), did not differ between the experienced yoga practitioners and controls (Figures 1C,D). Vertex analysis revealed no group differences in the shape of the hippocampus, caudate nucleus, or thalamus.

#### Brain Activation During Working Memory Subcomponent Processes

A main effect of encoding among all participants was observed in four clusters spanning the left (cluster size: 722 voxels; MNI coordinates of maximum *z*-value in mm: X −52, Y 0, Z 48) and right middle frontal and precentral gyri (cluster size: 635 voxels; MNI coordinates of maximum *z-value* in mm: X 44, Y −2, Z 54), the paracingulate gyrus (cluster size: 492 voxels; MNI coordinates of maximum *z-value* in mm: X −4, Y 4, Z 58), and the bilateral lateral occipital cortex (cluster size: 1805 voxels; MNI coordinates of maximum *z-value* in mm: X −18, Y −96, Z −11).

**Figure 2:**
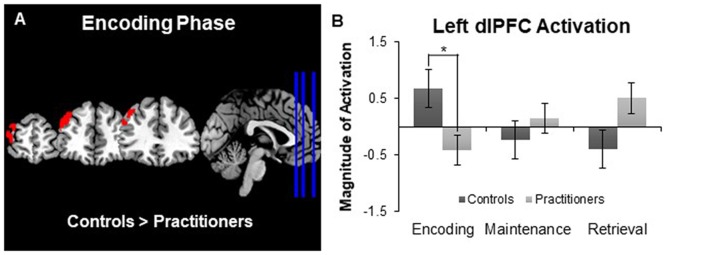
Panel **(A)** depicts group differences in the encoding vs. baseline contrast map. Panel **(B)** shows the magnitude of activation during the Sternberg working memory task between groups. Experts showed less activation in the left dorsolateral prefrontal cortex than controls during the encoding phase, but not the maintenance or retrieval phases, of the Sternberg working memory task. *p < 0.05.

The between groups analysis (using a *Z* statistic threshold of 2.3 and a corrected cluster threshold of *p* = 0.05) revealed one cluster in the left dorsolateral prefrontal cortex in which activation during encoding was significantly lower for the practitioners than for the controls (cluster size: 655 voxels; MNI coordinates of maximum z-value in mm: X −42, Y 46, Z 30; see Figure 2A). By creating a mask of this area we were able to extract the average contrast of parameter estimate values in this area for each participant. The bar graph in Figure 2B displays the average contrast of parameter estimate values within each group. It appears that while controls display more activation in the dorsolateral prefrontal cortex during encoding compared to baseline, the experienced yoga practitioners display less activation in this same region. We did not observe any brain areas in which activation during encoding was significantly greater for practitioners than controls. Furthermore, we did not find any differences in activation in either direction between practitioners and controls during maintenance or retrieval.

## Discussion

This study examined the similarities and differences in brain structure and function between 13 experienced yoga practitioners and age- and sex-matched controls. We found a significant difference in the left hippocampal volume, where experienced yoga practitioners exhibited larger gray matter volume than control participants. Additionally, during the performance of the Sternberg working memory task, experienced yoga practitioners exhibited less activation in the left dorsolateral prefrontal cortex region than controls. Together, these findings contribute to our limited understanding of the neurological correlates of yoga practice.

Differences in gray matter volume have been reported in previous yoga studies. Froeliger B. E. et al. (2012) and Froeliger B. et al. (2012) found yoga meditation practitioners (*N* = 7, females = 6, mean age = 36.4 years) to have significantly higher gray matter volume in a number of regions including the left para-hippocampal gyrus, hippocampus and insula. Our findings also corroborate with the evidence from a yoga-based intervention study that examined the effects of a 6-month yoga intervention on cortical structures among seven healthy older adults (Hariprasad et al., 2013) (age range 69–81 years., males = 4). Their results also showed a significant increase in bilateral hippocampus gray matter volume. The hippocampus is known to be critically involved in learning and memory processes (Squire, 1992). Yoga effects on hippocampal volume are also aligned with findings from the erobic exercise (Erickson et al., 2011) and mindfulness literature (Hölzel et al., 2011). Future research needs to examine the underlying mechanisms, other cortical and subcortical regions, and the similarities and differences within the different forms of exercise (such as yoga vs. erobic) that lead to similar neurobiological effects.

In addition to examining the subcortical volumes, we compared activation during the performance of the Sternberg working memory task between the two groups. During the encoding phase of the task we observed activation in the middle frontal, precentral and paracingulate gyri for all of the participants, which is in line with previous studies (Bedwell et al., 2005). However, there was a significant difference in the activation of the left dorsolateral prefrontal cortex. Experienced yoga practitioners exhibited less activation in this region than the controls. The dorsolateral prefrontal cortex is a region that is typically activated during the encoding phase of verbal working memory tasks (Bedwell et al., 2005) and may be sensitive to increasing load during encoding, such that this area is engaged more as task load is increased (Rypma and D’Esposito, 1999). Less dependency on recruiting the dorsolateral prefrontal cortex to perform the task may be reflective of increased efficiency by experienced yoga practitioners. This is in line with behavioral studies that suggest yoga practice has a beneficial influence on working memory performance (Gothe et al., 2014; Gothe and McAuley, 2015). However, because the task load was relatively low during all trials, it is not surprising that we did not observe any objective differences in task performance as determined by the accuracy and reaction time of participants. Future studies should strive to use tasks with a higher cognitive load so that the association between task performance and brain measures can be elucidated. It is also important to note that the behavioral performance of the two groups was the same, in spite of significant differences observed in the left hippocampal volume.

Several functions of the hippocampus and dorsolateral prefrontal cortex can be implicated in the practice of yoga. Both of these regions are involved in the modulation of cortical arousal and emotional regulation (Milad et al., 2007). Yoga practice is holistic and involves a combination of physical exercises, breathing and meditation that include relaxation and yoga has been shown to have psychological effects including decreased anxiety (Kirkwood et al., 2005) and stress (Chong et al., 2011). In addition to improvements in mood and anxiety, imaging studies have also shown yoga interventions to increase thalamic gamma aminobutyric acid levels in healthy young adults (Streeter et al., 2010). Preliminary evidence also suggests that yoga has a down regulating effect on both the sympathetic nervous system and the hypothalamic-pituitary adrenal axis in response to stress (Ross and Thomas, 2010). More recently, salivary cortisol and self-reported affect were found to mediate the relationship between yoga practice and improvement in behavioral measures of cognitive performance, specifically working memory and mental flexibility (Gothe et al., 2016). It appears that regular yoga practice may result in optimal regulation of affect and emotion for the practitioner, which may result in the effective activation of the dorsolateral prefrontal cortex as observed in the present study.

Because of the cross-sectional nature of the study our results should be interpreted as tentative. Although this small sample size may have impacted our power and undermined our ability to detect some of the effects of yoga, we were able to recruit and test twice as many participants compared to previous studies. Given the pilot nature of this study, we were limited in our measurements and chose to examine the subcomponents of working memory based on the preliminary studies and literature. Future studies should examine whether the effects of yoga practice are selective in impacting working memory, or also influence other executive functions and corresponding brain regions. While our groups were well matched on age, sex and education levels, future studies could also include a measure of intelligence and account for menopausal status for female participants as it has been recently shown to affect functional connectivity and hippocampal volume (Lisofsky et al., 2015). It is possible that differences in cerebral blood flow could underlie the differences we observed in the dorsolateral prefrontal cortex, as such an effect had been shown in the aging (Moses et al., 2014) and cognitive training literature(Chapman et al., 2013). Nevertheless, there are a number of strengths to this study worth noting. Contrary to previous studies (Froeliger B. E. et al., 2012; Froeliger B. et al., 2012; Hariprasad et al., 2013), we also accounted for differences in physical activity levels and cardiorespiratory fitness between the study groups, using an established measure of self-reported physical activity as well as an objective assessment of cardiorespiratory fitness. Importantly, these characteristics did not differ between the yoga and control groups, indicating that the observed differences in brain structure and function can be attributed to differences in yoga practice specifically rather than to exercise or fitness levels of the study participants. Another strength of the present study is its specific focus on working memory, as opposed to a measuring cognition as a congregate of memory, attention, processing speed and other executive functions. It is important to make a distinction between the subcomponent processes of cognitive functions, as they are likely to recruit differential brain resources and regions. The differences we observed in encoding but not in the maintenance or retrieval phases of the working memory task demonstrate the value of investigating working memory as well as other executive functions at their subcomponent levels. While yoga practice may not exhibit differences on overall task performance, specific brain functions (such as encoding alone) may show differential brain activation or patterns as observed in our study. Finally, our sample characteristics were different from previous studies, where researchers have recruited yoga meditation practitioners, i.e., participants with a significant meditation and/or mindfulness practice (Froeliger B. E. et al., 2012; Froeliger B. et al., 2012). Although the practice of yoga involves meditation, the postures and breathing exercise are just as important in Hatha yoga styles of practice. Our sample of experienced yoga practitioners primarily reported the practice of yoga postures (66% of their yoga practice time) as compared to previous studies where the samples were categorized as yoga meditation practitioners.

In conclusion, the present study contributes to the topical field of yoga and cognition and to our understanding of the neurobiological correlates of yoga practice. The regions affected by yoga practice, i.e., the hippocampus and the prefrontal cortex, also show significant age related changes (Grady, 2012; Toga, 2015). Therefore, behavioral interventions like yoga may hold promise to mitigate age-related and neurodegenerative declines. Systematic randomized trials of yoga based exercise, as well as long term longitudinal studies on yoga practitioners, are needed to identify the extent and scope of cognitive and neurobiological changes and their underlying mechanisms that occur as a function of yoga practice.

## Public Significance

The field of physical activity has extensively examined the effects of exercise, particularly aerobic training on cognition and executive functions. However, compared to this extensive body of work, far fewer scientific studies have examined movement-based embodied contemplative practices such as yoga. To our knowledge, this is the first neuroimaging study examining differences in brain activation among experienced yoga practitioners and controls during the performance of an executive function based working memory task.

## Author Contributions

NG: study conceptualization, execution and data management. JH and CT: recruitment and data collection. JD: study conceptualization, MRI image sequencing and data processing.

## Conflict of Interest Statement

The authors declare that the research was conducted in the absence of any commercial or financial relationships that could be construed as a potential conflict of interest.
